# Phosphorylation at S153 as a Functional Switch of Phosphatidylethanolamine Binding Protein 1 in Cerebral Ischemia-Reperfusion Injury in Rats

**DOI:** 10.3389/fnmol.2017.00358

**Published:** 2017-10-31

**Authors:** Zhong Wang, Jiyuan Bu, Xiyang Yao, Chenglin Liu, Haitao Shen, Xiang Li, Haiying Li, Gang Chen

**Affiliations:** Nerve Research Laboratory, Department of Neurosurgery and Brain, The First Affiliated Hospital of Soochow University, Suzhou, China

**Keywords:** cerebral ischemia-reperfusion injury, phosphatidylethanolamine binding protein 1, phosphorylation, apoptosis, inflammation, rats

## Abstract

This study aimed to estimate the role of phosphatidylethanolamine binding protein 1 (PEBP1) in cerebral ischemia-reperfusion (I/R) injury and the underlying mechanisms. Middle cerebral artery occlusion/reperfusion (MCAO/R) model in adult male Sprague Dawley rats (250–280 g) were established and cultured neurons were exposed to oxygen-glucose deprivation/reoxygenation (OGD/R) to mimic I/R injury *in vitro*. Expression vectors encoding wild-type PEBP1 and PEBP1 with Ser153Ala mutation (S153A), PEBP1 specific siRNAs, and human recombinant PEBP1 (rhPEBP1) were administered intracerebroventricularly. Endogenous PEBP1 level and its phosphorylation at Ser153 were increased within penumbra tissue and cultured neurons after I/R, accompanied by decreased interaction between PEBP1 and Raf-1. There was a trend toward increased Raf-1/MEK/ERK/NF-κB signaling pathway and phosphatidylcholine-phospholipase C (PC-PLC) activity after I/R, which was enhanced by wild-type PEBP1overexpression and rhPEBP1 treatment and inhibited by PEBP1 (S153A) overexpression. And PEBP1 (S153A) overexpression increased its interaction with Raf-1, reduced infarct size, neuronal death and inflammation, and improved neurological function after I/R, while wild-type PEBP1overexpression exerted opposite effects, suggesting that phosphorylation at Ser153 may exert as a functional switch of PEBP1 by switching PEBP1 from Raf-1 inhibition to PC-PLC activation following I/R. Compared with PEBP1 knockdown, PEBP1 (S153A) overexpression exerted a better rescue effect on I/R injury, which further proved that PEBP1 may be a good protein gone bad with phosphorylation at S153 as a functional switch following I/R. Collectively, our findings suggest that PEBP1 contributed to neuronal death and inflammation after I/R. Selective inhibition of PEBP1 phosphorylation may be a novel approach to ameliorate I/R injury.

## Introduction

Stroke is a devastating disease that can cause cognitive and motor dysfunction, and even acute death, and is becoming the leading cause of mortality and morbidity worldwide (Chen et al., 2014; Wang et al., 2016). In this study, we focus on ischemic stroke, which makes up more than 85% of all strokes (Fagan, 2010). As neuronal death starts as early as 5 min after oxygen deprivation (Radermacher et al., 2012), early recanalization is currently the preferred treatment strategy for ischemic stroke (Radermacher et al., 2012; Imam et al., 2016). However, it is unfavorable that, ischemia/reperfusion (I/R)-induced brain injury leads to unsatisfactory clinical outcome of the therapy (Molina, 2011; Min et al., 2015; Dang et al., 2016). Although there are a lot of researches have focused on the prevention and treatment of cerebral I/R injury (Horn and Schlote, 1992; Cai et al., 2016), the exact mechanisms underlying the I/R injury still remain elusive (Liu et al., 2016; Mandeville et al., 2016).

Bernier and Jolles (1984) first found and isolated a 23 kDa cytosolic protein from bovine brain. Due to its potential of binding to phospholipid, the protein was named as phosphatidylethanolamine binding protein (PEBP) (Bernier et al., 1986). At present, a class of proteins with the similar function is put together as a PEBP family, including more than 400-member proteins from bacteria to human (He et al., 2016). Among them, PEBP1 was found the ability of binding to Raf-1 and inhibiting the downstream signaling pathways of Raf-1, and then PEBP1 earned another name as Raf-1 kinase inhibitory protein (RKIP) (Yeung et al., 1999). PEBP1 has been found to have crucial roles in neural development and differentiation (Ojika et al., 1992) and participate in many neurological disorders, including Alzheimer’s disease, depression and nervous system tumors (Danzer, 2012; Amemori et al., 2015).

It is generally accepted that inflammatory responses and apoptosis play crucial roles in the cascade reaction of cerebral I/R injury, and the Raf-1/MEK/ERK/nuclear factor-κB (NF-κB) signaling pathway is an important mechanism of both inflammatory responses and apoptosis (Jean et al., 1998; Kokubo et al., 2002). The interaction between PEBP1 and Raf-1 has been reported to inhibit the transduction of Raf-1/MEK/ERK/NF-κB signaling pathway, which inhibited inflammatory response and apoptosis subsequently (Mayo et al., 2001; Karin and Greten, 2005). Moreover, protein kinase C (PKC), which is activated under I/R condition (Hafeez et al., 2014), can phosphorylate PEBP1 at serine 153 (Corbit et al., 2003), and the phosphorylated PEBP1 was reported to inhibit the interaction between PEBP1 and Raf-1 (Corbit et al., 2003). PEBP1 also has been identified as a binding partner of phosphatidylcholine-phospholipase C (PC-PLC), which also plays important roles in inflammatory responses and apoptosis (Li et al., 2010). The interaction of PEBP1 with PC-PLC could positively regulate PC-PLC activity, negatively regulate autophagy and participate in atherosclerosis development (Wang et al., 2013). Accumulating evidence views autophagy as an adaptive response to combat against stressful circumstances, including I/R condition. And PC-PLC has been shown to contribute to subarachnoid hemorrhage-induced early brain injury in our previous study (Li et al., 2015). Where does PEBP1 go after release from Raf-1 due to PEBP1 phosphorylation, and whether PEBP1 phosphorylation switches PEBP1 from Raf-1 to other binding partners, such as PC-PLC, are still unclear.

In conclusion, accumulating researches suggested a therapeutic potential of PEBP1 in cerebral I/R injury. However, until now, no study has investigated the contribution of PEBP1 to I/R injury, and no study has been performed on the effects of PEBP1 phosphorylation, especially at ser153, on Raf-1/MEK/ERK/NF-κB signaling pathway, PC-PLC activation, and autophagy pathway during I/R. Therefore, the aim of this study was to investigate the role of PEBP1 and to assess the therapeutic potential of PEBP1 in cerebral I/R injury.

## Materials and Methods

### Experimental Animals

All experiments were approved by the Ethics Committee of the First Affiliated Hospital of Soochow University and were in accordance with the guidelines of the National Institutes of Health on the care and use of animals. Adult male Sprague–Dawley (SD) rats weighing 250–280 g were purchased from Animal Center of Chinese Academy of Sciences, Shanghai, China. The rats were housed in temperature- and humidity-controlled animal quarters with a 12-h light/dark cycle.

### Establishment of Experimental Middle Cerebral Artery Occlusion/Reperfusion (MCAO/R) Model in Rats

**FIGURE 1:**
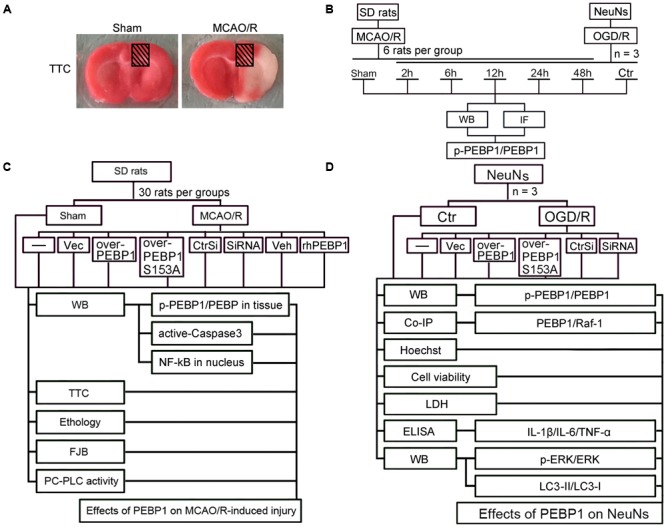
Middle cerebral artery occlusion/reperfusion (MCAO/R) model and Experimental Design. **(A)** TTC staining of brain coronal sections at 2 h after MCAO/R surgery. **(B)** Time course analysis of the protein levels of PEBP1 and phosphorylated PEBP1 (p-PEBP1) after I/R. **(C)** Roles of PEBP1 in MCAO/R-induced injury. **(D)** Mechanisms underlying PEBP1 actions during I/R. p-PEBP1: phosphorylated PEBP1 at Ser153; PEBP1 (S153A): PEBP1 with Ser153 mutated to alanine.

The MCAO/R model was performed as previously reported (Li et al., 2014). Briefly, focal cerebral ischemia in SD rats was realized by right-sided endovascular MCAO under the operating microscope. First, the common, internal, and external carotid arteries (CCA, ICA, and ECA) were exposed through the midline cervical incision. Then, a piece of filament (Cinontech, Beijing, China) was inserted into the right CCA and advanced it along the right ICA until the tip occluded the proximal stem of the middle cerebral artery. A heating pad was used to maintain the rectal temperature between 36.5 and 37.5°C. After 2 h of ischemia, the filament was withdrawn for reperfusion. Sham controls only exposed the blood vessels through the midline cervical incision. After the surgery, brain tissue samples were achieved separately for 2, 3, 5-triphenyltetrazolium chloride (TTC) staining (**Figure 1A**). In this model, around ischemic core, there is a penumbra (peri-infract) region, which could be rescued (Ashwal et al., 1999; Sarabi et al., 2008). The penumbra region was shown in shade in **Figure 1A**. This study focuses on the role of PEBP1 in this penumbra region.

### Cell Culture

As described previously (Zhai et al., 2016), primary neurons from SD rats were obtained and cultured. Whole brains of 17th-day rat embryos were used for preparing cell culture. Every effort was made to reduce the number of embryos used and their suffering. First, we removed blood vessels and the meninges. Then, the brains were digested with trypsin for 5 min. After that, we centrifuged the brain suspension at 500 × *g* for 5 min and inoculated neurons in 6-well and 12-well plates in neurobasal medium (GIBCO, Carlsbad, CA, United States). Neurons were maintained in a 5% CO_2_ atmospheric incubator at 37°C. We renewed half of the medium every 2 days for 2 weeks. Finally, the neurons were exposed to the indicated treatments and harvested.

### Establishment of *in Vitro* Oxygen-Glucose Deprivation/Reoxygenation (OGD/R) Model

Briefly, neurobasal medium was replaced with DMEM (GIBCO, Carlsbad, CA, United States) and cells were transferred to a 5% CO_2_ and 95% N_2_ atmospheric incubator for 2 h at 37°C. After that, neurons were cultured in neurobasal medium again and maintained in 5% CO_2_ atmospheric incubator for indicated time periods. Control groups were cultured in neurobasal medium in 5% CO_2_ atmospheric incubator for the same period. The pH of culture medium was maintained at 7.2.

### Experiment Grouping

#### Part One: Time Course Analysis of the Protein Levels of PEBP1 and Phosphorylated PEBP1 (p-PEBP1) after I/R

As shown in **Figure 1B**, *in vivo*, 36 SD rats (41 SD rats were used, 36 SD rats were survived after surgery) were randomly assigned to six groups of six SD rats each: a sham group and five experimental groups arranged by reperfusion time: 2, 6, 12, 24, and 48 h. The brain penumbra of six SD rats in each group was achieved at the indicated time point and used for western blot analysis and double immunofluorescence analysis. *In vitro*, experiment was performed to evaluate the effect of OGD/R treatment on the level of p-PEBP1 and PEBP1 in cultured neurons.

#### Part Two: Effects of PEBP1 on Cerebral I/R Injury

As shown in **Figure 1C**, *in vivo*, 270 SD rats (294 rats were used, 270 rats were survived) were randomly assigned to nine groups (*n* = 30 per group): sham group, MCAO/R group, MCAO/R + GFP-vector group, MCAO/R + GFP-PEBP1 group, MCAO/R + GFP-PEBP1 (S153A) group, MCAO/R + control-siRNA group, MCAO/R + PEBP1-siRNA group, MCAO/R + vehicle group, and MCAO/R + human recombinant PEBP1 (rhPEBP1, 15 μg/kg body weight) group. The transfection of plasmids and siRNAs was given at 48 h before surgery and the injection of rhPEBP1 was given immediately following reperfusion intracerebroventricularly. Based on the previous time course study, 6 SD rats per group were extracted for western blot analysis of the level of p-PEBP1 and PEBP1 at 6 h after MCAO/R surgery. 10 SD rats were sacrificed at 12 h after MCAO/R surgery and used for western blot analysis of intranuclear accumulation of NF-κB p65 subunit and PC-PLC activity assay. Another 14 SD rats per group were examined for behavioral impairment and sacrificed at 72 h after MCAO/R surgery. Among them, 8 SD rats per group were used for TTC staining, 6 SD rats per group were used for fluoro-jade B (FJB) staining and western blot analysis of the level of active-caspase 3. The dose of rhPEBP1 was chosen based on the preliminary experiment results, which showed that 15 μg/kg body weight is the most cost-efficient dose of rhPEBP1 to induce a significant increase in the protein level of PEBP1 in brain tissues at 24 h after MCAO/R surgery (Supplementary Figure 1).

#### Part Three: Mechanisms Underlying PEBP1 Actions during I/R

As shown in **Figure 1D**, cultured neurons were divided into seven groups: control group, OGD/R group, OGD/R + GFP-vector group, OGD/R + GFP-PEBP1 group, OGD/R + GFP-PEBP1 (S153A) group, OGD/R + control-siRNA group and OGD/R + PEBP1-siRNA group. The transfection of plasmids and siRNAs were given at 48 h before OGD/R. Based on the previous time course study, at 6 h after reperfusion, we performed western blot analysis and co-immunoprecipitation analysis to test the level of p-PEBP1 and PEBP1 and the interaction between PEBP1 and Raf-1. At 12 h after reoxygenation, western blot analysis was performed to test the level of Raf-1/MEK/ERK/NF-κB signaling pathway and autophagy. At 24 h after reoxygenation, sulforhodamine B (SRB) assay and hoechst 33258 staining were performed to test cell viability and neuronal apoptosis, and all the culture supernatants were collected for lactate dehydrogenase (LDH) activity assay and enzyme-linked immunosorbent assay (ELISA) to evaluate neuronal necrosis and inflammatory response.

### Antibodies and Drugs

Rabbit anti-p-PEBP1 (phospho S153) antibody (ab75971), rabbit anti-PEBP1 antibody (ab76582), rabbit anti-GFP antibody (ab6556), rabbit anti-Histone H3 antibody (ab8580), rabbit anti-NeuN antibody (ab177487) and mouse anti-NeuN (1B7) antibody (ab104224) were from Abcam (Cambridge, MA, United States). Mouse anti-PEBP1(D-5) antibody (sc-365973), rabbit anti-NF-κB p65 (C20) antibody (sc-372), rabbit anti-p-ERK 1/2 antibody (sc-23759-R), mouse anti-ERK 1/2 (MK1) antibody (sc-135900), mouse anti-β-actin (C4) antibody (sc-47778) and mouse anti-GAPDH (G-9) antibody (sc-365062) were from Santa Cruz Biotechnology (Santa Cruz, CA, United States). Mouse monoclonal anti-Raf-1/c-Raf antibody (R2404) was from Sigma–Aldrich Corporation (Merck, German). Rabbit anti-LC3B antibody (2775) and rabbit anti-cleaved-Caspase3 antibody (9661) were from Cell Signaling Technology (Cell Signaling Technology, Inc., BOS, United States). Normal mouse IgG (sc-2025) and normal rabbit IgG (sc-2027) were from Santa Cruz Biotechnology. Secondary antibodies for western blot analysis, including goat anti-rabbit IgG-HRP (sc-2004) and goat anti-mouse IgG-HRP (sc-2005), were from Santa Cruz Biotechnology. Secondary antibodies for immunofluorescence microscopy, including Alexa Fluor-555 donkey anti-rabbit IgG antibody (A31572), Alexa Fluor-488 donkey anti-rabbit IgG antibody (A21206), Alexa Fluor-555 donkey anti mouse IgG antibody (A31570) and Alexa Fluor-488 donkey anti-goat IgG antibody (A11055) were from Invitrogen. rhPEBP1 (PRO-722) was from ProSpec-Tany (Israel). PKC inhibitor (sc-3007) was from Santa Cruz Biotechnology (Santa Cruz, CA, United States).

### Western Blot Analysis

The brain homogenate collected from SD rats and the cultured neurons were lysed in RIPA lysis buffer (P0013; Beyotime, Shanghai, China). We centrifuged the samples at 12,500 × *g* for 15 min at 4°C, collected the supernatants, and determined the protein concentration of the supernatants by standard BCA (P0012; Beyotime, Shanghai, China) method. Then, the samples (100 μg/lane) were loaded onto a 15% SDS polyacrylamide gel, separated, and electrophoretically transferred to a polyvinylidene difluoride membrane (IPVH00010; Millipore, Billerica, MA, United States). The membrane was blocked in 5% bovine serum albumin (BSA, BIOSHARP, Hefei, AH, China) for 2 h at room temperature. Subsequently, the membrane was further incubated with the primary antibody overnight at 4°C and then incubated with HRP-conjugated secondary antibodies for 2 h at room temperature. The membrane was revealed with the enhanced chemiluminescence kit (Beyotime Institute of Biotechnology) and the relative quantities of proteins were analyzed with Image J software.

To test the level of p-PEBP1 by western blot easily, we enriched PEBP1 from total proteins by immunoprecipitation using PEBP1 antibody before electrophoresis.

### Nuclear Protein Extraction

The nuclear content of NF-κB was tested by western blot assay of nuclear protein. Nuclear protein extraction was performed using a Nuclear and Cytoplasmic Protein Extraction Kit from Beyotime (P0027).

### Immunofluorescence Microscopy

The brain samples were fixed in 4% paraformaldehyde, embedded in paraffin, cut into 4 μm sections. The cultured neurons were fixed in 4% paraformaldehyde. Then, the sections and neurons were incubated with primary antibodies and secondary antibodies. Normal mouse IgG and normal rabbit IgG were used as negative controls for the immunofluorescence assay. The sections and neurons were observed in a fluorescence microscope (OLYMPUS BX50/BX-FLA/DP70; Olympus Co., Japan) or a laser scanning confocal microscope (ZEISS LSM 880, Carl Zeiss AG, Germany). The relative fluorescence intensity was analyzed with the Image J program. The quantitative analysis was performed by an observer who was blind to the experimental group.

### Construction of Expression Plasmids and Site Directed Mutagenesis

The coding region of rat PEBP1 cDNA was sub-cloned into pGFP-N2 expression vector to produce the pGFP-N2-PEBP1 construct. In addition, a rat PEBP1 cDNA construct with a mutation in a possible key phosphorylation site (Ser153) was prepared. S153A mutant (Ser153 of PEBP1 were mutated to alanine) was also sub-cloned into the pGFP-N2 expression vector as the wild-type PEBP1 cDNA, which allowed us to measure their location by fluorescence assay. All of the constructs were confirmed by DNA sequencing.

### siRNAs

Specific siRNAs against PEBP1 were obtained from Ribobio. The knockdown efficiency of all the three siRNAs was separately tested by *in vitro* transfection and western blot analysis. The most efficient one was used in the following study.

PEBP1 siRNA sequences:

(1) Sense: 5′ CCCACCCAGGTCATGAATA dTdT 3′

Antisense: 3′ dTdT GGGTGGGTCCAGTACTTAT 5′

(2) Sense: 5′ GCACTGTCCTCTCCGAATA dTdT 3′

Antisense: 3′ dTdT CGTGACAGGAGAGGCTTAT 5′

(3) Sense: 5′ GCAACAAGTCTGGAGACAA dTdT 3′

Antisense: 3′ dTdT CGTTGTTCAGACCTCTGTT 5′

### Transfection

Cultured neurons were transfected with indicated expression vectors using Lipofectamine^®^3000 Transfection Reagent (Invitrogen, L3000-015) or siRNAs using Lipofectamine RNAi MAX (Invitrogen, 13778-075) according to the manufacturer’s instructions. At 48 h after transfection, neurons were performed OGD/R treatment. Then, the cultured neurons were harvested and analyzed.

### Immunoprecipitation Analysis

Immunoprecipitation analysis was performed as described previously (Dang et al., 2015). First, the brain and cell samples were lysed in RIPA lysis buffer (P0013; Beyotime, Shanghai, China). For immunoprecipitation analysis, the lysate was incubated with specific antibodies or normal IgG (negative control) overnight at 4°C with rotary agitation. Then, Protein A+G Sepharose beads were added to each immune complex and the mixture was incubated for 4 h at 4°C with rotary agitation. SDS-PAGE and immunoblotting were performed for further protein separation and detection.

### Hoechst 33258 Staining

Neurons were fixed in 4% paraformaldehyde for 10 min, stained by Hoechst 33258 at room temperature for 15 min, and observed by a fluorescence microscope (OLYMPUS BX50/BX-FLA/DP70; Olympus Co., Japan). Normal neuron nuclei were homogeneously stained blue, while the nuclei of apoptotic neurons displayed nuclear fragmentation or chromatin condensation. Apoptosis was assessed in ≥300 cells. The quantitative analysis was performed by an observer who was blind to the experimental group.

### Cell Viability

Cell viability was examined by SRB assay. After indicated treatments, neurons were fixed with 50% trichloroacetic acid and stained with a SRB solution. Finally, SRB was measured with a Bio-Rad Micro plate reader. The cell viability was performed in triplicate and repeated three independent times at least.

### Lactate Dehydrogenase (LDH)

The concentration of LDH in medium was determined with LDH assay kit (A020-2; Jiancheng Biotech, Nanjing, China). These assays were performed according to manufacturer’s instructions, and these data were expressed relative to standard curves prepared for them.

### Enzyme-Linked Immunosorbent Assay (ELISA)

The concentrations of IL-1β, IL-6, and TNF-α in medium were determined by corresponding ELISA kits (R&D Systems Inc., United States). These assays were performed according to manufacturer’s instructions, and these data were expressed relative to standard curves prepared for them.

### TTC Staining

After indicated treatments, brains were removed and frozen in -20°C for 10 min, and sectioned coronally into 2 mm thick slices starting from the frontal pole. The olfactory bulb and cerebellum were discarded. Then, the brain slices were immersed in TTC solution (D025; Jiancheng Biotech, Nanjing, China) for 15–30 min at 37°C. After staining, the brain slices were washed three times and captured with a digital camera. To calculate the infarct volume, first, we calculated the total mean infarct area of each section as the average of the infarct area on the rostral and the caudal side. Then, we calculate the total area by adding the average area from each section and calculate the infarct volume as the multiplication of the total area by 2 mm (thickness of the sections). At last, the infarct volume was expressed as a percentage of the ipsilateral hemispheric volume.

### FJB Staining

The Brain sections were deparaffinized, rehydrated, and incubated in 0.06% K permanganate for 15 min. Then, the brain sections were rinsed in deionized water, immersed in 0.001% FJB working solution (0.1% acetic acid) for 30 min, and dried in incubator (50–60°C) for 10 min. The brain sections were cleared in xylene, cover slipped with 1 drop of mountant and observed by a fluorescence microscope (OLYMPUS BX50/BX-FLA/DP70; Olympus Co., Japan).

### Neurological Impairment

As described previously (Dang et al., 2015), at the 72 h after MCAO/R surgery, SD rats were examined for behavioral impairment with a scoring system and monitored for activity, appetite and neurological defects.

### PC-PLC Activity Assay

According to the manufacturer’s protocol as described previously (Wang et al., 2013), the PC-PLC activity in the cultured neurons was examined with the Amplex Red PC-PLC-specific assay kit (A12218, Molecular Probes).

### Statistical Analysis

Graph pad prism 7.00 was used for all statistical analysis. Neurobehavioral scores were shown as median with interquartile range. All the other data are presented as mean ± SD. Frequency distribution for the neurobehavioral score assay. One-way analysis of variance followed by either a Dunnett’s or a Tukey’s *post hoc* test, the former for comparisons to a single control group, the latter to compare across multiple groups. *p* < 0.05 was considered to be significant difference.

## Results

### General Observation

No significant changes in blood pressure, body temperature and heart beat were detected in any of the experimental groups (Supplementary Figure 2). The mortality rate of rats in the sham group was 0% (0/36 rats), and it was 9.7% (29/299 rats) in the MCAO/R group.

### I/R Increased the Protein Level and the Phosphorylation of PEBP1 at ser153 and Inhibited the Interaction between PEBP1 and Raf-1 in Neurons

**FIGURE 2:**
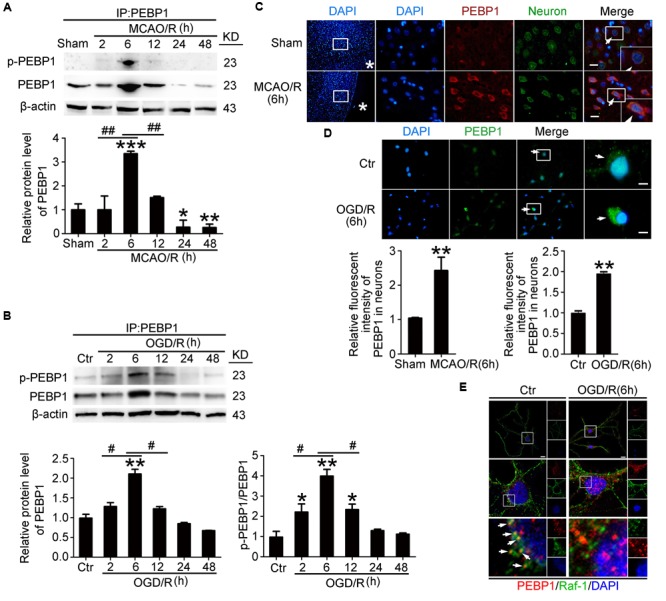
Effects of I/R on the protein level, phosphorylation and the interaction with Raf-1 of PEBP1. In **(A,B)**, to test the level of p-PEBP1 by western blot easily, immunoprecipitation (IP) using PEBP1 antibody was performed to enrich PEBP1 from total proteins before electrophoresis. **(A)** Western blot analysis and quantification of the level of PEBP1 and p-PEBP1 in penumbra tissue. ^∗^*p* = 0.0107 vs. sham group, ^∗∗^*p* = 0.0097 vs. sham group, ^∗∗∗^*p* < 0.001 vs. sham group, 2 h group vs. 6 h group ^##^*p* = 0.0085, 12 h group vs. 6 h group ^##^*p* = 0.0091 (*n* = 6/group). **(B)** Western blot analysis and quantification of the level of PEBP1 and p-PEBP1 in cultured neurons. Phosphorylation levels were evaluated by the ratio of p-PEBP1 to total PEBP1. In PEBP1, ^∗∗^*p* = 0.0089 vs. control group, 2 h group vs. 6 h group ^#^*p* = 0.023, 12 h group vs. 6 h group ^#^*p* = 0.013 (*n* = 6/group). In p-PEBP1, 2 h group vs. control group ^∗^*p* = 0.0123, 12 h group vs. control group ^∗^*p* = 0.0101, ^∗∗^*p* = 0.0011 vs. control group, 2 h group vs. 6 h group ^#^*p* = 0.018, 12 h group vs. 6 h group ^#^*p* = 0.023 (*n* = 6/group). **(C)** Double immunofluorescence analysis was performed with antibodies for PEBP1 (red) and neuron marker (NeuN, green). Nuclei were fluorescently labeled with DAPI (blue). Representative images of the sham and MCAO/R (6 h) groups are shown. Asterisks mark the outside of the brain. Arrows indicate the distribution of PEBP1 in neurons. Scale bar = 20 μm. The relative fluorescent intensity of PEBP1 in neurons was shown below. ^∗∗^*p* = 0.0091 vs. sham group (*n* = 6/group). **(D)** Immunofluorescence analysis was performed with antibody for PEBP1 (green) in cultured neurons. Nuclei were fluorescently labeled with DAPI (blue). Representative images of control group and OGD/R (6 h) group were shown. Arrows indicate the distribution of PEBP1 in neurons. Scale bar = 20 μm. The relative fluorescent intensity of PEBP1 in neurons was shown below. ^∗∗^*p* = 0.0085 vs. control group (*n* = 6/group). Statistical comparisons between groups were performed using one-way analysis of variance followed by either a Dunnett’s or a Tukey’s *post hoc* test, the former for comparisons to a single control group, the latter to compare across multiple groups. **(E)** Double immunofluorescence analysis was performed with antibodies for PEBP1 (red) and Raf-1 (green). Nuclei were fluorescently labeled with DAPI (blue). Representative images of the control and OGD/R (6 h) groups are shown. Arrows indicate the co-localization of PEBP1 and Raf-1 in neurons. Scale bar = 20 μm. p-PEBP1: phosphorylated PEBP1 at Ser153.

To detect the expression and phosphorylation of PEBP1 after MCAO/R, through western blot analysis of protein samples from penumbra tissue, we first tested the protein level of PEBP1 and p-PEBP1 (ser153) in the penumbra tissue. The results demonstrated that, compared with the sham group, both the protein level of PEBP1 and p-PEBP1 (ser153) in the penumbra tissue reached the highest point at 6 h, and then rebounded gradually (**Figure 2A**). Consistent with the *in vivo* data, western blot assay showed that the protein levels of PEBP1 and p-PEBP1 (ser153) in cultured neurons were significantly increased by OGD/R at 2–12 h and peaked at 6 h (**Figure 2B**). Double immunofluorescence with the neuronal marker protein NeuN further verified the MCAO/R-induced increase in the protein level of PEBP1 in neurons (**Figure 2C**). Immunofluorescence staining of PEBP1 on cultured neurons treated with OGD/R stimulus *in vitro* also showed an OGD/R-induced increase in PEBP1 when compared with control group (**Figure 2D**). In addition, previous studies have indicated a critical role of PEBP1 phosphorylation at ser153 in PEBP1 binding properties (Corbit et al., 2003). We then performed double immunofluorescence in cultured neurons at 6 h after OGD/R and found that, compared with control group, there was a significant decrease in the co-location between PEBP1 and Raf-1, which means a weakening of the interaction between PEBP1 and Raf-1 (**Figure 2E**).

In addition, PKC have been identified as responsible for the phosphorylation of PEBP1 at serine 153 (Corbit et al., 2003), while PKC is activated under I/R condition (Hafeez et al., 2014). To further study the mechanism underlying the increased phosphorylation of PEBP1 in neurons following I/R, we tested the effects of a PKC inhibitor (Santa cruz, sc-3007) on the phosphorylation at S153 of PEBP1 in cultured neurons exposed to OGD/R (Supplementary Figure 3). The results showed that, compared with OGD/R group, PKC inhibition significantly decreased the phosphorylation at S153 of PEBP1 in cultured neurons exposed to OGD/R, suggesting that the increased phosphorylation at S153 of PEBP1 following I/R is mediated by PKC, at least partially.

### OGD/R-Induced Phosphorylation of PEBP1 at ser153 Inhibited the Interaction between PEBP1 and Raf-1 in Cultured Neurons

**FIGURE 3:**
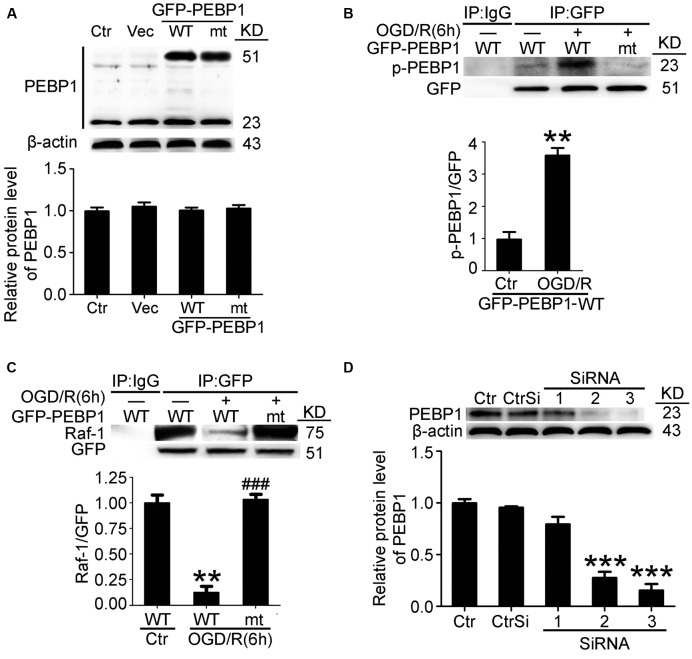
Transfection efficiency of PEBP1 expression vectors and siRNAs and the effect of mutation of serine153 to alanine on PEBP1 phosphorylation and the interaction between PEBP1 and Raf1 in cultured neurons. **(A)** Neurons were separately transfected with GFP-N2 expression vector (Vec), GFP- wide type PEBP1 (WT) and GFP-PEBP1 (S153A) (MT). Neurons were harvested at 48 h after transfection. Equal amounts of indicated cell lysate proteins were immunoblotted using PEBP1 antibody. The quantitative analysis of the protein level of PEBP1 was shown below and there were no significant changes (*n* = 6/group). **(B)** Western blot and quantitative analysis of the level of p-GFP-PEBP1 in neurons. To test the level of p-GFP-PEBP1 easily, immunoprecipitation (IP) using GFP antibody was performed to enrich GFP-PEBP1 from total proteins before electrophoresis. ^∗∗^*p* = 0.0025 vs. control + GFP-PEBP1-WT group (*n* = 6/group). **(C)** GFP-PEBP1/Raf-1 interaction was determined using IP. Quantitative histogram analysis was performed. ^∗∗^*p* = 0.0012 vs. control + GFP-PEBP1-WT group, ^###^*p* < 0.001 vs. OGD/R + GFP-PEBP1-WT group (*n* = 6/group). **(D)** PEBP1 silencing efficiency. ^∗∗∗^*p* < 0.001 vs. control siRNA (CtrSi) group (*n* = 6/group). Statistical comparisons between groups were performed using one-way analysis of variance followed by either a Dunnett’s or a Tukey’s *post hoc* test, the former for comparisons to a single control group, the latter to compare across multiple groups. p-PEBP1: phosphorylated PEBP1 at Ser153; WT: wild type; MT: PEBP1 with Ser153 mutated to alanine.

To further elucidate the role of phosphorylation at Ser153 in PEBP1 functions, expression vectors encoding rat wild-type PEBP1 and PEBP1 with Ser153Ala mutation were prepared. First, both wild type GFP-PEBP1 and the mutant protein were strongly expressed in transfected neurons (51 KD), and exogenous expression of PEBP1 did not affect the level of endogenous PEBP1 (23 kD) (**Figure 3A**). Notably, compared with that in endogenous PEBP1 (**Figures 2B,E**), OGD/R exerted a similar induction of the phosphorylation of GFP-PEBP1 at Ser153 and inhibition of the interaction between GFP-PEBP1 and Raf-1, which was almost abolished by the mutation of Ser153 to Ala (**Figures 3B,C**), therefore supporting Ser153 as a key site for OGD/R-induced PEBP1 phosphorylation.

In addition, a time course analysis of the levels of GFP-PEBP1 and phosphorylated GFP-PEBP1after OGD/R were also tested (Supplementary Figure 4). Consistent with the trends of endogenous PEBP1, both GFP-PEBP1 and p-GFP-PEBP1 (ser153) protein level in the penumbra tissue also decreased gradually after 6 h of reperfusion.

Finally, the interference efficiency of three different siRNAs specific for PEBP1 was tested, and the most efficient one (siRNA-3) was used in the following study (**Figure 3D**).

### Exogenous Expression of PEBP1 and Exogenous rhPEBP1 Treatment Increased the Level of p-PEBP1 in Penumbra Tissue after MCAO/R

**FIGURE 4:**
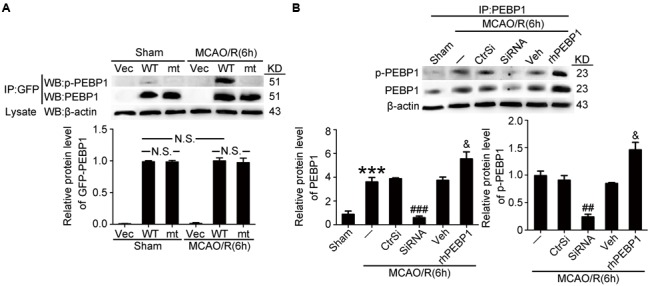
Transfection efficiency of PEBP1 expression vectors and siRNAs and the effect of rhPEBP1 on PEBP1 phosphorylation in penumbra tissue at 6 h after MCAO/R conditions. **(A)** Immunoprecipitation (IP) of tissue lysates with GFP antibody to enrich GFP-PEBP1 from total proteins. Western blots of IP with indicated antibodies showed the transfection efficiency of PEBP1 expression vectors and the phosphorylation of wide type GFP-PEBP1 and GFP-PEBP1 (S153A). There were no significant changes between the protein level of wide type GFP-PEBP1 and GFP-PEBP1 (S153A) both in sham group (*p* = 0.381) and MCAO group (*p* = 0.408) (*n* = 6/group). **(B)** IP of tissue lysates with PEBP1 antibody to enrich PEBP1 from total proteins. Western blot analysis and quantification of the level of PEBP1 and p-PEBP1. Phosphorylation levels were evaluated by the ratio of p-PEBP1 to β-actin. In PEBP1, ^∗∗∗^*p* < 0.001 vs. sham group, ^###^*p* < 0.001 vs. MCAO/R + control-siRNA group, ^&^*p* = 0.035 vs. MCAO/R + vehicle group. In p-PEBP1, ^##^*p* = 0.0031 vs. MCAO/R + control-siRNA group, ^&^*p* = 0.029 vs. MCAO/R + vehicle group (*n* = 6). Statistical comparisons between groups were performed using one-way analysis of variance followed by either a Dunnett’s or a Tukey’s *post hoc* test, the former for comparisons to a single control group, the latter to compare across multiple groups. p-PEBP1: phosphorylated PEBP1 at Ser153; WT: wild type; MT: PEBP1 with Ser153 mutated to alanine.

Then, we tested the transfection efficiency of exogenous expression vectors *in vivo* by western blot analysis. The results demonstrated that both wild type GFP-PEBP1 and the mutant protein were strongly expressed in transfected penumbra tissue (**Figure 4A**). In addition, compared with the sham group, a significant increase in the levels of PEBP1 and p-PEBP1 (ser153) was observed in MCAO/R group, which was attenuated by PEBP1 knockdown and exacerbated by exogenous rhPEBP1 treatment (**Figure 4B**).

### Phosphorylation at S153 Exerts as a Functional Switch of PEBP 1 in Neurons under OGD/R Stimulus

**FIGURE 5:**
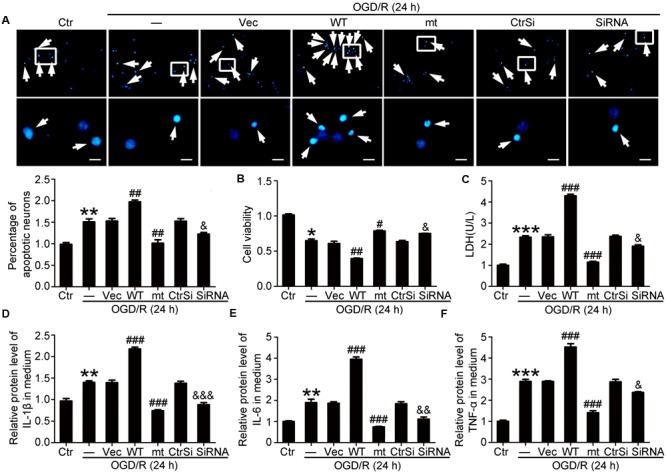
Effects of PEBP1 overexpression and knockdown on neuron apoptosis and inflammatory response in cultured neurons exposed to OGD/R for 24 h. **(A)** Hoechst 33258 Staining. Arrows indicated the nuclei of apoptotic neurons. Scale bar = 20 μm. ^∗∗^*p* = 0.0013 vs. control group, ^##^*p* = 0.0031 in OGD/R + wide type GFP-PEBP1 group and ^##^*p* = 0.003 in OGD/R + GFP-PEBP1 (S153A) group vs. OGD/R + vector group, ^&^*p* = 0.028 vs. OGD/R + control-siRNA group (*n* = 3). **(B)** Cell viability analysis. The mean value for control group was normalized to 1.0. ^∗^*p* = 0.0323 vs. control group, ^##^*p* = 0.0042 vs. OGD/R + vector group, #*p* = 0.024 vs. OGD/R + vector group, ^&^*p* = 0.028 vs. OGD/R + control-siRNA group (*n* = 3). **(C)** LDH analysis. ^∗∗∗^*p* < 0.001 vs. control group, ^###^*p* < 0.001 vs. OGD/R + vector group, ^&^*p* = 0.018 vs. OGD/R + control-siRNA group (*n* = 3). **(D–F)** ELISA assay of the contents of IL-1β, IL-6 and TNF-α in culture supernatant. In IL-1β, ^∗∗^*p* = 0.0054 vs. control group, ^###^*p* < 0.001 vs. OGD/R + vector group, ^&&&^*p* < 0.001 vs. OGD/R + control-siRNA group (*n* = 3). In IL-6, ^∗∗^*p* = 0.0043 vs. control group, ^###^*p* < 0.001 vs. OGD/R + vector group, ^&&^*p* = 0.0035 vs. OGD/R + control-siRNA group (*n* = 3). In TNF-α, ^∗∗∗^*p* < 0.001 vs. control group, ^###^*p* < 0.001 vs. OGD/R + vector group, ^&^*p* = 0.036 vs. OGD/R + control-siRNA group (*n* = 3). In **(A–F)**, the mean values for control group were normalized to 1.0. Statistical comparisons between groups were performed using one-way analysis of variance followed by either a Dunnett’s or a Tukey’s *post hoc* test, the former for comparisons to a single control group, the latter to compare across multiple groups. WT: wild type; MT: PEBP1 with Ser153 mutated to alanine.

We then investigated the function of PEBP1 in OGD/R-induced neuronal injury *in vitro*. First, TUNEL staining showed that only a few TUNEL-positive apoptotic cells were observed in the control group, while the apoptotic index was found to be significantly higher in the OGD/R group (**Figure 5A**). As compared with the OGD/R group, the apoptotic index was significant exacerbated by wild type GFP-PEBP1 overexpression and attenuated by GFP-PEBP1 (S153A) overexpression, suggesting phosphorylation at Ser153 may be a functional switch of PEBP1 in OGD/R-induced neuronal injury. Cell viability assay also identified the rescue effects of the Ser153Ala mutation in PEBP1 in neurons under OGD/R stimulus (**Figure 5B**). Consistently, the necrosis index showed the same trend with that in the apoptotic index (**Figure 5C**). Finally, inflammatory cytokines, including IL-1β, IL-6, and TNF-α, were found to be significantly higher in the culture supernatant of the OGD/R group than that in the control group. Compared with OGD/R group, the mean inflammatory cytokine contents were higher in wild type GFP-PEBP1 overexpression group and lower in GFP-PEBP1 (S153A) overexpression group (**Figures 5D–F**). In addition, *in vitro* rescue effects of PEBP1 knockdown on OGD/R-induced neuronal injury were also detected (**Figure 5**).

### Phosphorylation at Ser153 Exerts as a Functional Switch of PEBP1 in MCAO/R-Induced Brain Injury in Rats

**FIGURE 6:**
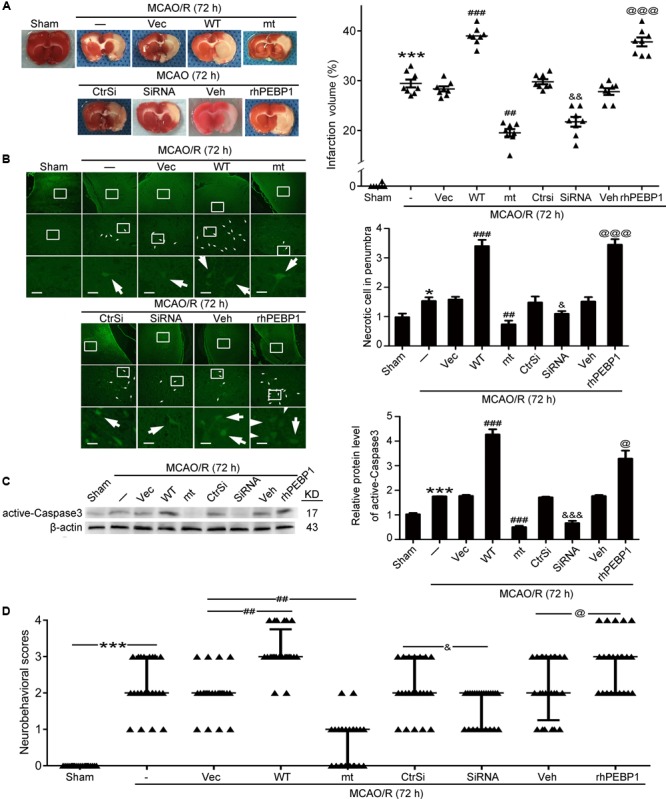
Effects of PEBP1 overexpression and knockdown and rhPEBP1 treatment on brain injury at 72 h after MCOA/R surgery. **(A)** TTC staining analysis and the quantification of the infarct volume. The mean values for MCAO/R group were normalized to 1.0. ^∗∗∗^*p* < 0.001 vs. sham group, ^###^*p* < 0.001 vs. MCAO/R + vector group, ^##^*p* = 0.0037 vs. MCAO/R + vector group, ^&&^*p* = 0.0021 vs. MCAO/R + control-siRNA group, ^@@@^*p* < 0.001 vs. MCAO/R + vehicle group (*n* = 8). **(B)** FJB staining. Arrows indicated FJB-positive neurons. Scale bar = 60 μm. ^∗^*p* = 0.011 vs. sham group, ^###^*p* < 0.001 vs. MCAO/R + vector group, ^#^*p* = 0.038 vs. MCAO/R + vector group, ^&&^*p* = 0.0045 vs. MCAO/R + control-siRNA group, ^@@@^*p* < 0.001 vs. MCAO/R + vehicle group (*n* = 6). **(C)** Western blot analysis and quantification of the protein level of active-Caspase3. ^∗∗∗^*p* < 0.001 vs. sham group, ^###^*p* < 0.001 vs. MCAO/R + vector group, ^&&&^*p* < 0.001 vs. MCAO/R + control-siRNA group, ^@^*p* = 0.034 vs. MCAO/R + vehicle group (*n* = 6). **(D)** Clinical behavior scores. ^∗∗∗^*p* < 0.001 vs. sham group, ^#^*p* = 0.044 vs. MCAO/R + vector group, ^&&^*p* = 0.0099 vs. MCAO/R + control-siRNA group, ^@^*p* = 0.044 vs. MCAO/R + vehicle group (*n* = 14). In **(B,C)**, the mean values for sham group were normalized to 1.0. Statistical comparisons between groups were performed using one-way analysis of variance followed by either a Dunnett’s or a Tukey’s *post hoc* test, the former for comparisons to a single control group, the latter to compare across multiple groups. WT: wild type; MT: PEBP1 with Ser153 mutated to alanine.

First, TTC staining displayed the benefit of PEBP1 (S153A) overexpression and PEBP1 knockdown on the infarct volume, while wild type GFP-PEBP1 overexpression and exogenous rhPEBP1 treatment exerted opposite effects (**Figure 6A**). To further examine the role of PEBP1 in MCAO/R-induced brain injury, FJB staining, caspase 3 activation and clinical behavior scores were tested. Compared to the sham group, FJB-positive cells were increased in the penumbra tissue in the MCAO/R group, which was significantly attenuated by PEBP1 (S153A) overexpression and PEBP1 knockdown and enhanced by wild type GFP-PEBP1 overexpression and exogenous rhPEBP1 treatment (**Figure 6B**). Consistently, western blot assay of caspase 3 activation showed the same trend (**Figure 6C**). Finally, clinical behavior scores assay showed the rescue effects of PEBP1 (S153A) overexpression and PEBP1 knockdown on the neurobehavioral function of rats subjected to MCAO/R, while wild type GFP-PEBP1 overexpression and exogenous rhPEBP1 treatment exerted opposite effects (**Figure 6D**).

### Phosphorylation of PEBP1 at Ser153 Disrupts Its Inhibition of Raf-1/ERK1/2/NF-κB Signaling Pathway

**FIGURE 7:**
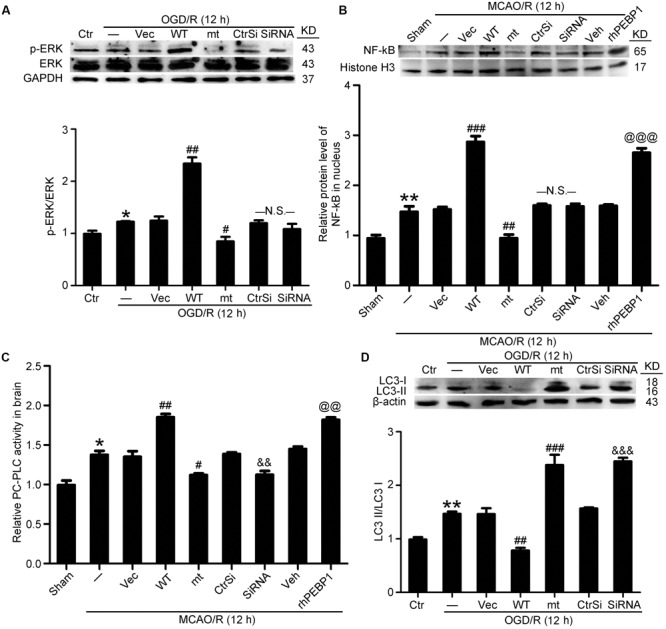
Effects of PEBP1 overexpression and knockdown and rhPEBP1 treatment on ERK/NF-κB signaling pathway, PC-PLC activity and autophagy after I/R at indicated times. **(A)** Western blot analysis and quantification of the level of p-ERK and ERK in cultured neurons exposed to OGD/R for 12 h. ^∗^*p* = 0.076 vs. control group, ^##^*p* = 0.0033 vs. OGD/R + vector group, ^#^*p* = 0.046 vs. OGD/R + vector group, N.S. = no significant (*n* = 6). **(B)** Western blot analysis and quantification of the level of NF-κB in nuclear protein of penumbra tissue at 12 h after MCAO/R. ^∗∗^*p* = 0.0056 vs. sham group, ^###^*p* < 0.001 vs. MCAO/R + vector group, ^###^*p* = 0.0055 vs. MCAO/R + vector group, ^@@@^*p* < 0.001 vs. MCAO/R + vehicle group, N.S. = no significant (*n* = 6). **(C)** The relative PC-PLC activity in penumbra tissue at 12 h after MCAO/R. ^∗^*p* = 0.014 vs. sham group, ^##^*p* = 0.0042 vs. MCAO/R + vector group, ^#^*p* = 0.011 vs. MCAO/R + vector group, ^&&^*p* = 0.0078 vs. MCAO/R + control-siRNA group, ^@@^*p* = 0.039 vs. MCAO/R + vehicle group (*n* = 10). **(D)** Western blot analysis and quantification of the protein level of LC3 in cultured neurons exposed to OGD/R for 12 h. ^∗∗^*p* = 0.0055 vs. sham group, ^##^*p* = 0.0036 vs. MCAO/R + vector group, ^###^*p* < 0.001 vs. MCAO/R + vector group, ^&&&^*p* < 0.001 vs. MCAO/R + control-siRNA group (*n* = 6). In **(A,D)**, the mean values for control group were normalized to 1.0. In **(B,C)**, the mean values for control group were normalized to 1.0. Statistical comparisons between groups were performed using one-way analysis of variance followed by either a Dunnett’s or a Tukey’s *post hoc* test, the former for comparisons to a single control group, the latter to compare across multiple groups. WT: wild type; MT: PEBP1 with Ser153 mutated to alanine.

As shown in **Figures 2** and **3**, I/R-induced phosphorylation of PEBP1 at Ser153 inhibited the interaction between PEBP1 and Raf-1 neurons. To further investigate the mechanism whereby the PEBP1 action, the downstream of Raf-1pathway, including the phosphorylation of ERK1/2 and the nuclear transfer of NF-κB, was assessed by western blot analysis (**Figures 7A,B**). The results showed the phosphorylation level of ERK1/2 in neurons subjected to OGD/R to be significantly higher than that in the control group. Wild type GFP-PEBP1 overexpression and exogenous rhPEBP1 treatment significantly increased the level of ERK1/2 phosphorylation, while PEBP1 (S153A) overexpression exerted opposite effects (**Figure 7A**). Consistently, the nuclear content of NF-κB in MCAO/R group was significantly higher than that in the sham group, which was significantly attenuated by PEBP1 (S153A) overexpression and enhanced by wild type GFP-PEBP1 overexpression and exogenous rhPEBP1 treatment (**Figure 7B**). Notably, compared with OGD/R + control-siRNA group, PEBP1 knockdown did not induce significant changes in the phosphorylation of ERK1/2 and the nuclear transfer of NF-κB (**Figures 7A,B**).

### Phosphorylation of PEBP1 at Ser153 Promotes PC-PLC Activation and Inhibits Autophagy

As showed in **Figure 7C**, PC-PLC activity was significantly higher in MCAO/R group than that in the sham group. Compared with MCAO/R group, the PC-PLC activity was significantly enhanced in MCAO/R + PEBP1 group and MCAO/R + rhPEBP1 group, and weakened significantly in MCAO/R + PEBP1 (S153A) group and MCAO/R + PEBP1-siRNA group (**Figure 7C**). In addition, the results also revealed increases in the LC3-II/LC3-I conversion in OGD/R group, which was inhibited by wild type GFP-PEBP1 overexpression and exogenous rhPEBP1 treatment, while PEBP1 (S153A) overexpression and PEBP1 knockdown exerted opposite effects (**Figure 7D**).

## Discussion

Ischemic stroke is becoming the leading cause of mortality and morbidity worldwide with early recanalization as the preferred treatment strategy. However, I/R often involves unavoidable damages and leads to unsatisfactory clinical outcome of the therapy. At present, penumbra rescue by attenuating I/R injury is fully acknowledged and there is an urgent demand of efficient remedies for I/R injury. Using a clinically relevant rat MCAO/R model, we are exploring a new neuroprotective strategy targeting PEBP1. PEBP1 may be a good protein gone bad with phosphorylation at Ser153 as a functional switch following I/R. By attenuating neuronal death and inflammation, selective PEBP1 phosphorylation inhibition would limit I/R complications and benefit neurological outcomes.

**FIGURE 8:**
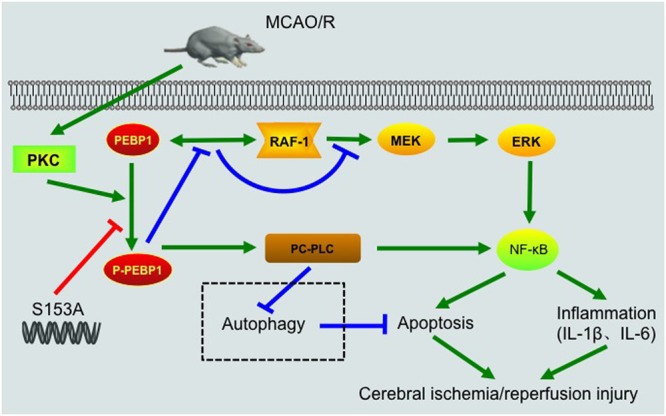
Schematic representations of potential mechanisms of PEBP1 in enhancing inflammatory response and neuron apoptosis after I/R injury. Under normal condition, PEBP1 binds to Raf-1 and inhibits the Raf-1/MEK/ERK/NF-κB signaling pathway indirectly. Following MCAO/R, I/R stimulus induces an increase in the protein of PEBP1 and activates PKC, which in turn promotes the phosphorylation of PEBP1. The phosphorylation of PEBP1 inhibits the interaction between PEBP1 and Raf-1, and relieves the Raf-1/MEK/ERK/NF-κB signaling pathway. Subsequently, the released p-PEBP1 from Raf-1 binds to another binding partner PC-PLC. The phosphorylated PEBP1 promotes PC-PLC activation, which further enhances NF-κB signaling pathway and regulates autophagy negatively. Finally, excessive NF-κB signaling and abnormal autophagy enhance the inflammatory response and neuron apoptosis, and aggravates cerebral I/R injury. The switch of PEBP1 from Raf-1 to PC-PLC was inhibited by mutation the Ser153 of PEBP1 to alanine.

Our present study showed the role of PEBP1 in the pathogenesis of cerebral I/R injury in a rat MCAO/R model. First, results showed elevated levels of PEBP1 and p-PEBP1 (p-ser53) in penumbra tissue after MCAO/R and cultured neuron suffered from OGD/R (**Figure 2**). In addition, there was a decrease in the interaction between PEBP1 and Raf-1 accompanied by the increase in the phosphorylation at ser153 of PEBP1 (**Figure 2**). Then, using site-directed mutagenesis of Ser153 to alanine in PEBP1, we found the adverse effect of wild type PEBP1 overexpression and the rescue effect of PEBP1 (S153A) overexpression on I/R-induced brain injury (**Figures 5, 6**), suggesting that the phosphorylation at Ser153 may exert as a functional switch of PEBP1. And, as previously reported (Corbit et al., 2003) and shown here, PEBP1 phosphorylation led to the activation of Raf-1 signaling (**Figure 7**). Finally, there was a trend toward increased Raf-1/MEK/ERK/NF-κB signaling pathway and PC-PLC activity after I/R with PEBP1 phosphorylation at Ser153 switching PEBP1 from Raf-1 inhibition to PC-PLC activation (**Figure 7**). The results also showed that, under I/R condition, PEBP1 knockdown did not further increase the Raf-1/MEK/ERK/NF-κB pathway, suggesting PEBP1 barely interacts with Raf-1 following I/R. By this binding partner switching, PEBP1 abolished its anti-inflammatory and anti-apoptotic effects, which is mediated by its binding to Raf-1, and obtained a pro-inflammatory and pro-apoptotic effect by binding to and activating PC-PLC. Notably, compared with PEBP1 knockdown, PEBP1 (S153A) overexpression seems to exert a better rescue effect on I/R-induced neuronal injury (**Figures 5, 6**), all which further proved that PEBP1 might be a good protein gone bad with phosphorylation at Ser153 as a functional switch following I/R (**Figure 8**).

This study focused on the PEBP1 phosphorylation status and its possible role in regulation of the interaction between PEBP1 and Raf-1 following I/R. As a commonly used tag for protein studies, GFP was fused with the exogenous expressed PEBP1 to distinguish the endogenous and exogenous PEBP1. Ideally, as shown in **Figures 2**–**4** and Supplementary Figure 4, under I/R condition, the phosphorylation and the binding to Raf-1 of PEBP1 and GFP-PEBP1 shared the same trend, suggesting that fusion with GFP did not induce unwanted side effects of PEBP1 in this study.

In addition, one protein level is regulated by both the expression and the degradation of the protein. As shown in **Figure 2**, I/R increased the protein levels of PEBP1 both in penumbra tissue and cultured neurons at 6 h after I/R, while I/R stimulus did not affect the protein level of GFP-PEBP1 at the time point (**Figures 3, 4**), suggesting that I/R condition increased the protein level of PEBP1 at a transcriptional level during 0–6 h after reperfusion. At the same time, PEBP1 phosphorylation reached a peak 6 h after reperfusion (**Figure 2**). After 6 h of reperfusion, there was a gradual decrease in both the level of PEBP1 and p-PEBP1 (ser153) (**Figure 2**). In order to explain the possible mechanisms underlying the decrease, we tested the changes of the level of GFP-PEBP1 and p-GFP-PEBP1 (ser153) after 6 h of reperfusion (Supplementary Figure 4). Consistent with the trends of endogenous PEBP1, both GFP-PEBP1 and p-GFP-PEBP1 (ser153) protein level in the penumbra tissue also decreased gradually after 6 h of reperfusion. The results suggested that there may be an endogenous degradation exerting a dominant regulation in PEBP1 protein level after 6 h of reperfusion. The degree of protein phosphorylation is regulated by phosphorylation/de-phosphorylation reaction. The decrease in the ratio of p-PEBP1 to total PEBP1 may be due to an increase in the de-phosphorylation after 6 h of reperfusion. Changes on Ser 153 phosphorylation of PEBP1 and whether the de-phosphorylation regulation is involved in PEBP1 protein level regulation under I/R condition need further investigation.

Beside as a cytosolic protein, PEBP1 also has been shown to be secreted into circulation and participates in cardiac physiology, such as atherosclerosis (Wang et al., 2013). So, PEBP1 as a secretory protein may interpret the adverse effect of rhPEBP1 shown in I/R injury (**Figure 6**). Most secretory proteins contain amino internal or terminal signal peptides that direct their sorting to endoplasmic reticulum (ER) and are transported to the plasma membrane or extracellular space via a process known as conventional secretion pathway or ER-Golgi secretory pathway (Deretic et al., 2012). However, bioinformatics analysis shows that PEBP1 protein does not contain signal peptides, which suggests that the conventional ER-Golgi secretory pathway is not suitable for PEBP1 secretion. Recent studies figure that, in mammalian systems, autophagy plays an unexpected role in the trafficking or secretion of secreted proteins (Liu et al., 2000). In other words, there is an unconventional secretion pathway, also termed “autosecretion,” in transporting the secretory proteins without signal peptides. As previously reported (Wang et al., 2013) and shown here, PEBP1 participates in the regulation of autophagy, suggesting a possibility of autosecretion of PEBP1. However, the exact mechanism underlying the PEBP1 secretion needs further study. In addition, as shown in **Figure 4B**, rhPEBP1 treatment induced an increase in the protein level of PEBP1 in penumbra tissue, which can be interpreted in the following two ways: recombinant protein got into brain cells, or rhPEBP1 treatment may induce a negative regulation mechanism in PEBP1 secretion. Due to the selective membrane permeability, the first explanation is very weak. Considering the negative regulation mechanism, rhPEBP1 treatment induces a high extracellular PEBP1 concentration and subsequently inhibits the extracellular secretion of the endogenous PEBP1 finally resulting in an increased PEBP1 protein level in penumbra tissue. However, the exact mechanism underlying the negative regulation needs further study.

In this study, we focused on the phosphorylation of PEBP1 and the possible role of p-PEBP1 in I/R injury. Beside phosphorylation, there is a slice of studies support the role of other post-translational modification like methylation in the expression of PEBP1 (Guo et al., 2013). Some study figured that the methylation of PEBP1 might decrease PEBP1 expression by the methylation of the promoter (Guo et al., 2013), while other study found that the loss of PEBP1 expression was not result from the promoter methylation (Martinho et al., 2009). Furthermore, the methylation under different backgrounds, different races of people and even different diseases, may have different effects on the PEBP1 expression. Changes on the methylation of PEBP1 and whether methylation is associated with the expression of PEBP1 after I/R injury need further investigation.

The current study has some limitations. First, our study used healthy adult male SD rats, which did not mimic human high-risk populations maximally, such as patients with cardiovascular diseases and the elderly. Second, in clinical, the most appropriate method of administration for the preventive treatment should be the oral route. However, taking into account the metabolism and absorption of PEBP1 in the gastrointestinal tract, we chose intracerebroventricular injection in this study. Third, besides binding to Raf-1 and PC-PLC, PEBP1 also could interact with many other signaling pathways, such as mitogen-activated protein kinase signaling pathway, G-protein-coupled receptor signaling pathway, and glycogen synthase kinase 3 beta signaling pathway (Rajkumar et al., 2016). In addition, PEBP1 is widely expressed in several organs and could be secreted in to circulation to participate in atherosclerosis (Wang et al., 2013). Therefore, the potential side effects of PEBP1 intervention on other organs, the interaction with other partners, and the serum content of PEBP1 should not be ignored. And as shown in **Figure 2A**, compared with sham group, there was a decrease in the protein level of PEBP1 at 24 and 48 h after MCAO/R. However, no significant change was tested between control group and OGD/R (24 h) group or OGD/R (48 h) group, suggesting there may be changes in the protein level of PEBP1 in other brain cell following I/R. In this study, we only focused on the role of PEBP1 in cerebral I/R injury via binding to Raf-1 and PC-PLC in neurons. Fourth, a critical gap in our knowledge of PC-PLC is the unavailability of its gene sequence and protein structure. As shown in this study, performing the activity assay has been an important strategy for the investigation of PC-PLC functions in mammalian cells (Li et al., 2010). Thus, the protein structure of PC-PLC and the mechanisms underlying the interaction between PEBP1 and PC-PLC need further investigation. Finally, the remarkable rescue effect of PEBP1 (S153A) overexpression suggests a powerful therapeutic potential of recombinant PEBP1 (S153A) protein, which we will study and explore further.

## Conclusion

As shown here for the first time, the essential role of phosphorylation status in regulating the function of PEBP1, and the remarkable increase in the level of PEBP1 and p-PEBP1 (p-Ser153) in penumbra tissue after MCAO/R, suggest that therapies targeting PEBP1 hold significant promise for the treatment and prevention of pathologic processes characterized by I/R injury.

## Author Contributions

GC and HL conceived and designed the study, including quality assurance and control. ZW and JB performed the experiments and wrote the paper. XY and CL designed the study’s analytic strategy. HS and XL helped conduct the literature review and prepare the Section “Materials and Methods” of the text. All authors read and approved the manuscript.

## Conflict of Interest Statement

The authors declare that the research was conducted in the absence of any commercial or financial relationships that could be construed as a potential conflict of interest.
