# Supplementary material for: Phosphorylation at S153 as a Functional Switch of Phosphatidylethanolamine Binding Protein 1 in Cerebral Ischemia-Reperfusion Injury in Rats
Source: Front Mol Neurosci. 2017 Oct 31;10:358. doi: 10.3389/fnmol.2017.00358 (PMC5671526; doi:10.3389/fnmol.2017.00358)
Supplement: Supplementary file 1 [file Data_Sheet_1.DOCX]

Supplementary Material

Phosphorylation at S153 as a functional switch of phosphatidylethanolamine binding protein 1 in cerebral ischemia-reperfusion injury in rats

Zhong Wang^#^; Jiyuan Bu^#^; Xiyang Yao; Chenglin Liu; Haitao Shen; Xiang Li; Haiying Li*; Gang Chen*

*** Correspondence:** Haiying Li or Gang Chen, nju_neurosurgery@163.com

## Supplementary Figures


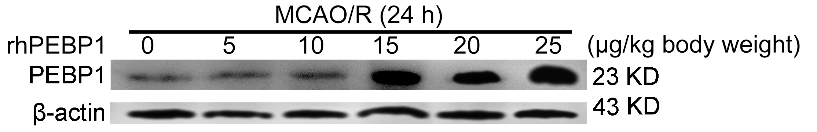


**Supplementary Figure 1.** Effects of exogenous rhPEBP1 treatment on the protein level of PEBP1 in penumbra tissue of MCAO/R rats. rhPEBP1 injection was performed immediately after reperfusion


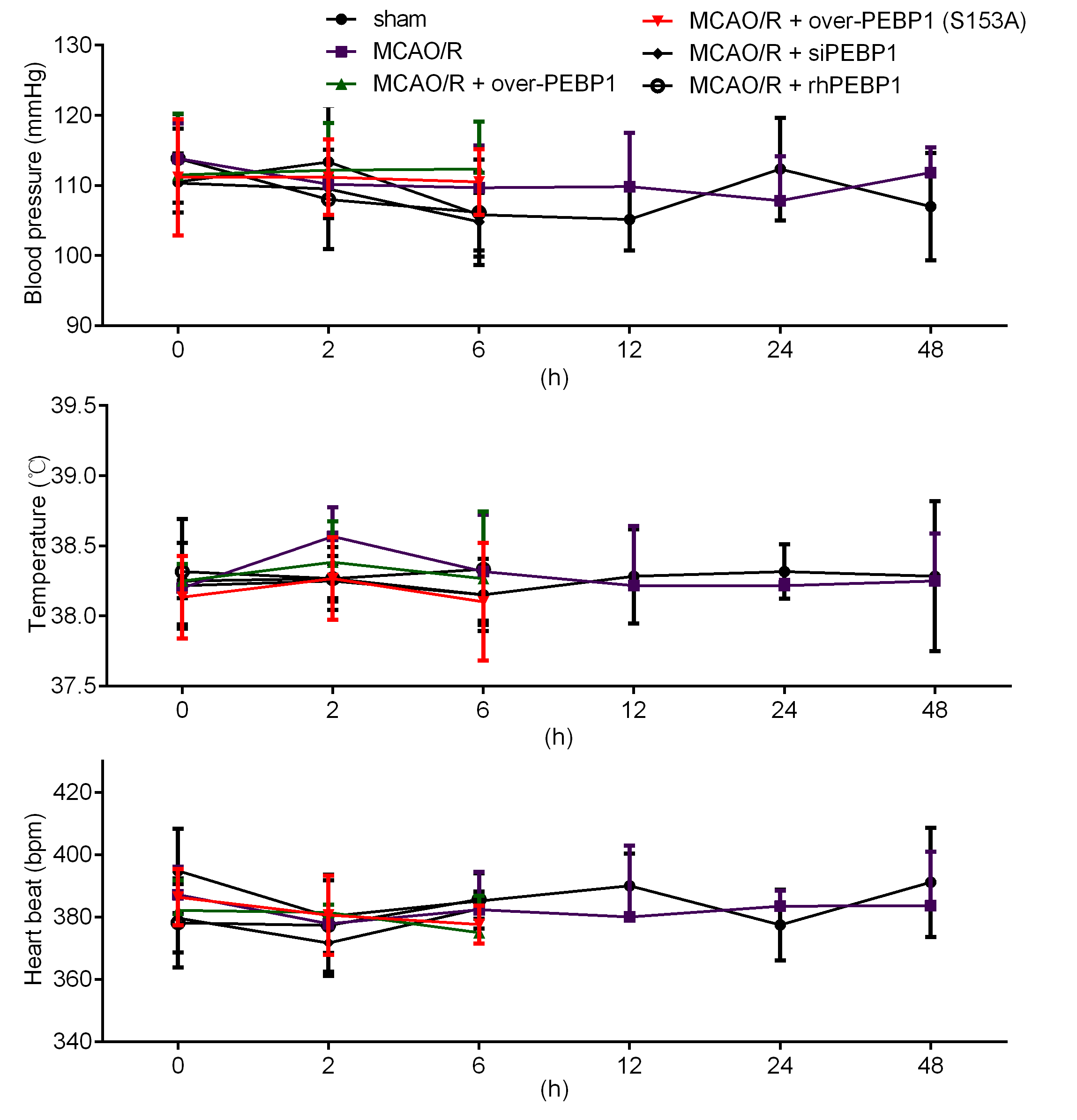


**Supplementary Figure 2.** Blood pressure, body temperature and heart beat of rats were monitored at indicated time points after MCAO/R onset. Data are means ± SD.





**Supplementary Figure 3.** Immunoprecipitation (IP) of cell lysates with PEBP1 antibody. Western blots of IP with p-PEBP1 (phospho S153) antibody and PEBP1 antibody. Mean values for control group were normalized to 1.0. Data are means ± SD. * * p < 0.01 vs. control group, ## p < 0.01 vs. OGD/R group, n = 3.


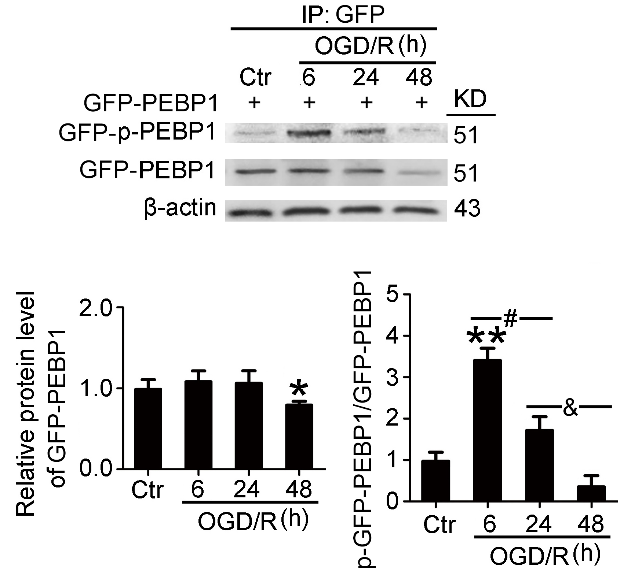


**Supplementary Figure 4.** Immunoprecipitation (IP) of cell lysates with GFP antibody to enrich GFP-PEBP1 from total proteins. Western blots of IP with p-PEBP1 (phospho S153) antibody and PEBP1 antibody showed the protein level of GFP-PEBP1 and the phosphorylation of GFP-PEBP1. Quantification of the level of GFP-PEBP1 and p-GFP-PEBP1 was shown. Mean values for control group were normalized to 1.0. Data are means ± SD. * p < 0.05, * * p < 0.01 vs. control group, # p < 0.01, ^&^ p < 0.01, n = 3.
